# Facial Asymmetry in a Crying Newborn: A Comparison of Two Cases and Review of Literature

**DOI:** 10.1155/2017/6368239

**Published:** 2017-02-26

**Authors:** Shreyas Arya, Sunil K. Jain, Carol J. Richardson

**Affiliations:** ^1^Department of Neonatal-Perinatal Medicine, Cincinnati Children's Hospital Medical Center, 3333 Burnet Ave, Cincinnati, OH 45229, USA; ^2^Department of Pediatrics/Division of Neonatology, University of Texas Medical Branch Galveston, Waverley Smith Pavilion, Room 6.104, 301 University Blvd., Galveston, TX 77555-0526, USA; ^3^Department of Pediatrics, Division of Neonatology, University of Texas Medical Branch Galveston, Research Building 6, Room 3.300, 301 University Blvd., Galveston, TX 77555-0587, USA

## Abstract

Facial asymmetry in a crying newborn can be due to a variety of different causes. Neonatal asymmetric crying facies (NACF) is a specific phenotype, which is often underrecognized. It is defined as asymmetry of the mouth and lips with grimacing or smiling, but a symmetric appearance at rest. NACF needs to be differentiated from complete facial palsy in a newborn, which can occur due to traumatic or developmental etiologies. Developmental causes can be present in isolation or may be a part of a recognized syndrome. While asymmetric lower lip depression may be seen in both conditions, complete facial palsy is also associated with upper and mid face deformities. We present a case of NACF and compare it to a case of facial palsy due to perinatal trauma. The purpose of this case series is to clarify some of the confusing nomenclatures and highlight the differences in the physical exam findings, diagnosis, and eventual prognosis of these cases.

## 1. Introduction

NACF represents a specific phenotype, the major symptom of which is the absence or weakness of the downward motion of the lateral side of the mouth with crying [1, 2]. This movement is carried out by a group of four facial muscles, the most important of which is the depressor anguli oris muscle (DAOM). All these muscles are innervated by branches of the facial nerve. The first case of facial asymmetry we describe was caused by the absence of DAOM. We compare this to a case of left-sided facial palsy caused by facial nerve injury secondary to perinatal trauma. The latter is an acquired condition and is also known as congenital facial paralysis. Facial palsy can also be seen due to errors in fetal development and is referred to as developmental facial paralysis [3]. This may be present in isolation or as part of recognized syndromes like Möbius, CHARGE, Goldenhar, hemifacial macrosomia, and so forth. A review of the aforementioned cases along with the relevant anatomy of the facial muscles and nerve will help the reader understand the subtle differences between these presentations.

## 2. Case Presentation

### 2.1. Case 1

**Figure 1:**
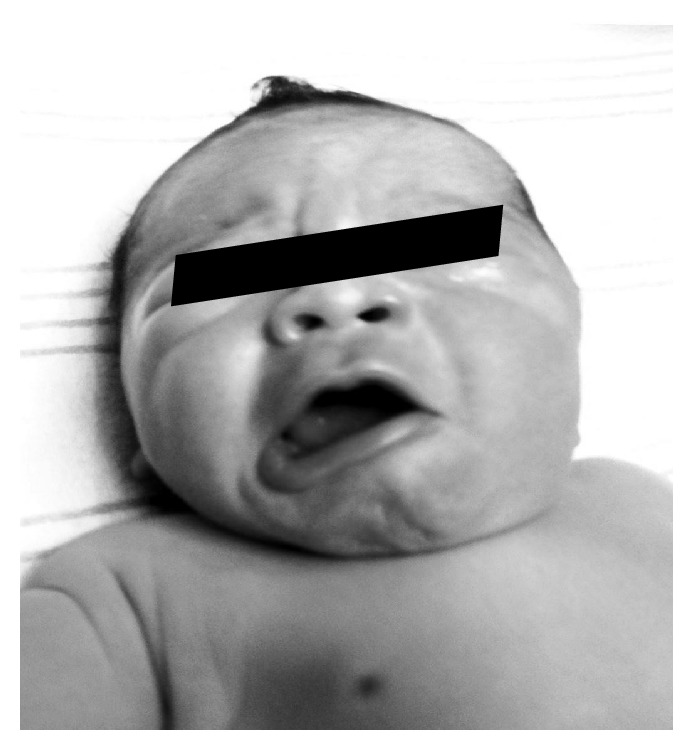
Neonatal asymmetric crying facies (NACF): asymmetry of face with deviation of mouth to right side with crying. Also note normal and symmetric nasolabial folds, forehead wrinkling, and eye closure bilaterally.

A male infant was born at 39 weeks of gestation to a gravida 3, para 3 mother with severe pregnancy induced hypertension. There was a history of marijuana use during pregnancy and the mother had a urinary tract infection, which was treated with Bactrim. The baby was born via an uncomplicated vaginal delivery and had Apgar scores of 8 and 9 at 1 and 5 minutes, respectively. Birth weight was 3860 grams. Asymmetry of the face with the deviation of mouth to the right side on crying was noted during the first few hours after birth. The baby had normal and symmetric nasolabial folds, forehead wrinkling, and eye closure bilaterally** (**Figure 1**)**. The baby was clinically diagnosed to have an absent DAOM on the left. A thorough cardiac evaluation did not reveal any abnormalities. He did not experience any difficulties with feeding in the postnatal period and was discharged from the hospital within 48 hours. The baby was followed by our team till 6 months of age, during which he continued to exhibit the deformity with crying.

### 2.2. Case 2

**Figure 2:**
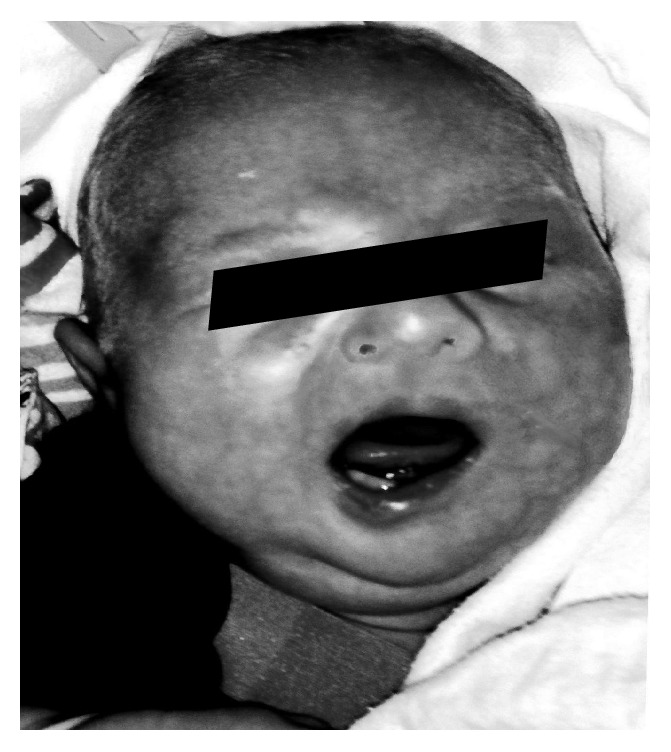
Facial nerve palsy: inability to close the left eyelid with deviation of the mouth to the right side with crying. Also note the less prominent nasolabial fold on the left.

A female infant was born at 35 weeks to a primigravida mother. Delivery was done by a Cesarean section due to breech presentation and fetal heart rate decelerations. The delivery was complicated by meconium staining and the baby had Apgar scores of 6 and 9 at 1 and 5 minutes, respectively. The infant also received surfactant for respiratory distress syndrome and required continuous positive airway pressure (CPAP) for respiratory support. The birth weight was 2420 g. A few hours following birth the baby was unable to close her left eyelid and there was a deviation of the mouth to the right side with crying. The nasolabial fold was also less prominent on the left side (Figure 2). The infant was clinically diagnosed to have facial nerve palsy on the left side. The patient was discharged with plans for close follow-up care by the pediatric ophthalmology team, due to inability to voluntarily close the left eyelid. At the 4-month follow-up visit, the mother reported significant improvement in left eye closure as well as the deviation of the mouth. The facial nerve palsy was noted to have completely resolved by 7 months of age.

## 3. Discussion

Facial asymmetry with crying in a newborn can be caused by a heterogeneous group of conditions, but our understanding of its etiologies has greatly improved in recent years. Neonatal asymmetric crying facies (NACF) is one such condition, which is often underrecognized. Even though the nomenclature might suggest that NACF is any asymmetry in facial appearance with crying, it is important to understand that it refers only to a specific phenotype characterized by absence or weakness of the downward motion of the lateral side of the mouth with crying (Figure 1) [2]. It is characterized by the asymmetry of mouth and lips with grimacing or smiling, but a symmetric appearance at rest. Many cases of NACF are isolated [4], but a study by Pasick et al. reported a 14% incidence of this condition in patients with 22q11.2 deletion, which is significantly higher than in the general population [5]. Association of NACF has also been described with congenital cardiac disease [6], neuroblastoma, mediastinal teratoma, and neurofibromatosis type I [1].

The downward motion of the lower lip is produced by the coordinated action of four muscles. The DAOM pulls the corner of the mouth downwards, laterally, and everts it. The depressor labii inferioris muscle (DLIM) extends from the mandible to the lower lip and depresses it. The mentalis muscle raises and protrudes the lower lip and the platysma muscle blends in with the DAOM and assists in its function. A majority of cases of NACF are caused by the hypoplasia of the DAOM and less commonly the DLIM. Injury to one of the peripheral branches of the facial nerve secondary to trauma may also present with this deformity in rare cases [7].

In Case 1, the downward and lateral movement with eversion of the lower lip is noted on the right but not on the left (Figure 1). In addition, this deformity did not resolve when followed over time. These findings along with the normal functioning of all other facial muscles are classical for the absence of DAOM. Hypoplasias of DAOM or DLIM give rise to a presentation also referred to as congenital unilateral lower lip palsy (CULLP) [8].

Though facial muscles can be seen in both computed tomography (CT) scan and magnetic resonance imaging (MRI), it is often difficult to obtain the necessary views. A CT scan also has the added risk of radiation to the neonate. Hence, Roedel et al. recommend using B-scan ultrasound for confirming the absence of facial muscles [9].

The facial nerve is the motor innervation of all facial muscles. The muscles of the upper face are represented bilaterally in the facial nerve nucleus of the pons, so a unilateral supranuclear facial nerve abnormality results in the contralateral weakness of all facial muscles except those involved in forehead wrinkling, closure of eye lids, and nostril dilatation with breathing. However, a lower motor neuron injury of the peripheral facial nerve will result in complete ipsilateral facial paralysis, without the sparing of muscles of the upper face, which is described in Case 2 (Figure 2). When this injury occurs in the peripartum setting, the deformity that results is known as congenital facial paralysis [3]. Falco and Eriksson followed the outcome of newborns with congenital facial paralysis and found that 89% eventually had a complete recovery [10]. The neonate described in Case 2 had significant improvement in his facial palsy by 4 months and complete resolution by 7 months of age.

In neonates, the mastoid process has not yet developed which makes the facial nerve vulnerable to injury as it exits through the stylomastoid foramen. Individual branches of the facial nerve, like the mandibular nerve, are especially prone to compression injury as it courses just above the lower edge of the mandible in a neonate. Trauma to the facial nerve may result from fetal positioning, as with compression injury from the shoulder in the intrauterine period, intrapartum pressure from the maternal pelvis, or forceps application [11]. Facial nerve palsy due to nerve compression is mostly an isolated finding and a search for other anomalies is not indicated [1].

Facial palsy can also be caused in newborns by developmental mishaps during the intrauterine life. These adverse events may be due to genetic factors, vascular events, and teratogenic insults which cause aplasia or hypoplasia of the cranial nerve nuclei, nuclear agenesis, and aplasia or hypoplasia of the facial nerve. The deformity that results is known as developmental facial paralysis and in contrast to congenital facial paralysis, it does not completely resolve with time. Developmental facial paralysis is frequently seen in association with genetic syndromes like Möbius, CHARGE, hemifacial microsomia, hereditary developmental facial paresis, and Goldenhar syndrome [3].

## 4. Conclusion

While the nomenclature might be confusing, neonatal asymmetric crying facies, congenital facial paralysis, and developmental facial paralysis all contribute to the dramatic presentation of facial deformity in a neonate. It is essential for practitioners to accurately diagnose these conditions and their most common associations. It is also important to be cognizant of the stigma that parents have in association with facial deformities. We hope that this article will facilitate a better understanding of this topic and aid physicians in early recognition, targeted screening, and counseling the parents of affected newborns.
